# The Impact of Hydrotherapy on Health‐Related Quality of Life, Pain and Mobility in Individuals With Mucopolysaccharidosis Type II (Hunter Syndrome): A Pilot Feasibility Study

**DOI:** 10.1002/jmd2.70114

**Published:** 2026-08-10

**Authors:** Andrew Oldham, Nicole Stockton, Mark Warburton, Calvin Heal, Marie Meehan, Karolina M. Stepien, the MPS Society

**Affiliations:** ^1^ Adult Inherited Metabolic Diseases Salford Royal Organisation, Northern Care Alliance NHS Foundation Trust Salford UK; ^2^ Metabolic Medicine Research Salford Royal Organisation, Northern Care Alliance NHS Foundation Trust Salford UK; ^3^ Centre for Biostatistics The University of Manchester Manchester UK; ^4^ Division of Cardiovascular Sciences University of Manchester Manchester UK

**Keywords:** HRQoL, hunter syndrome, hydrotherapy, MPSII, musculoskeletal morbidity, pain management

## Abstract

Enzyme Replacement Therapy (ERT) is the clinical standard for Mucopolysaccharidosis II (MPSII), yet its limited penetration into poorly vascularised tissues such as bone, cartilage and heart valves leaves participants with significant musculoskeletal morbidity. This small study evaluated the feasibility of hydrotherapy as an adjunct to ERT in addressing these therapeutic gaps and improving Health‐Related Quality of Life (HRQoL). Using a crossover design, the study assessed five male participants with MPSII. Key outcomes included HRQoL via the Short Form Health Survey (SF‐36), functional mobility by the 6‐Minute Walk Test (6MWT) and pain interference through the Brief Pain Inventory (BPI). Data from the SF‐36 and BPI showed improvements, including a mean improvement in Physical Functioning, Role Physical and Social Functioning and a mean reduction of 16.2 in pain interference. Analgesic relief over 24 h was 45.8% in the hydrotherapy arm, compared to 18% in the observation arm. Psychological conditions including anxiety, depression and kinesiophobia were also analysed. Participants demonstrated a mean increase of 44.5 m in the 6MWT, suggesting that the ‘unloading’ effect of water facilitated gait speed and endurance (compared to observation arm: mean difference 55.8 m 95% CI −51.6 to 163.1). Participants achieved a mean strength increase of 6.1 lb. during the hydrotherapy arm without adverse effects, corroborating the safety of aquatic resistance training in fragile populations. Our findings demonstrate the feasibility of hydrotherapy as an adjunct to ERT in participants with MPSII. By managing pain and improving HRQoL, hydrotherapy addresses physical and psychological burdens that ERT alone cannot achieve.

## Synopsis

This impact study indicates that hydrotherapy is a potential adjunct to ERT for patients with MPSII by using the physical properties of water to bridge therapeutic gaps in musculoskeletal function, pain interference and overall HRQoL.

## 1 Introduction

Mucopolysaccharidosis Type II (MPS II; OMIM 309900), commonly known as Hunter syndrome, is an X‐linked recessive lysosomal storage disorder with an estimated birth prevalence in Europe of 1 in 166 000 [1]. The condition is caused by mutations in the *IDS* gene, which result in a deficiency of the enzyme iduronate‐2‐sulfatase and the systemic accumulation of glycosaminoglycans (GAGs) [2].

Clinically, MPSII exists on a phenotypic continuum. It is broadly categorised into a severe form, characterised by progressive neurodegeneration, and an attenuated form, which lacks primary neuronal involvement [3]. Both forms share a diverse spectrum of somatic symptoms, including short stature, coarse facial features and significant skeletal and cardiopulmonary abnormalities [4]. Enzyme Replacement Therapy (ERT) is the gold standard for treatment, yet its limited penetration into tissues such as bone, cartilage and heart valves leaves participants with considerable musculoskeletal morbidity [5].

Chronic pain is a highly prevalent yet frequently under addressed symptom in the MPSII population, with reports suggesting it affects up to 94% of paediatric participants [6]. The pain syndromes associated with all MPS types are complex and multifactorial, typically presenting as a combination of neuropathic and musculoskeletal elements [4]. The primary driver of musculoskeletal discomfort is the progressive accumulation of GAGs, which leads to dysostosis multiplex (a specific pattern of skeletal deformities), persistent joint contractures and hip dysplasia [4, 7]. In addition to joint‐related issues, participants frequently suffer from neuropathic pain caused by physical nerve compression [8, 9]. Given the complexity of these pain profiles, management requires a multifaceted approach. While surgical decompression is often required for neuropathic issues, non‐surgical interventions such as physiotherapy have been strongly advocated for their ability to improve mobility and enhance the quality of life (QoL) [3].

Hydrotherapy involves specialised exercises in warm water supervised by a physiotherapist [10]. The properties of buoyancy, hydrostatic pressure and viscosity facilitate movements that are often restrictive or painful on land [11, 12, 13]. While anecdotal evidence suggests aquatic interventions reduce joint stiffness and pain while improving strength in MPS disorders [14, 15], quality evidence supporting its routine use remains limited.

The aim of this study is to assess the feasibility of hydrotherapy in the management of joint pain, mobility and quality of life in our participants with MPS type II.

## 2 Methods

### 2.1 Study Design

**FIGURE 1:**
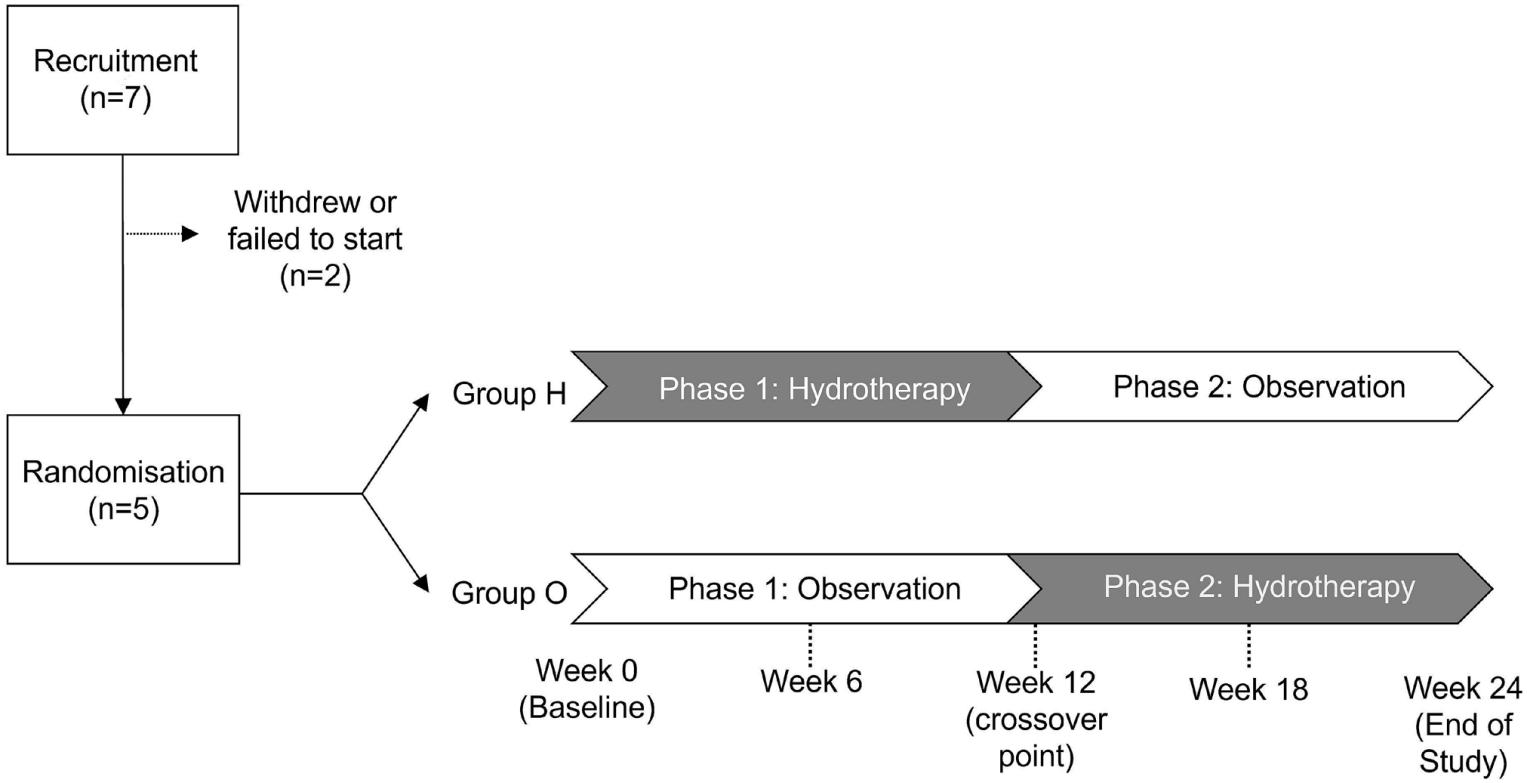
Study workflow. Group H (*n* = 3) completed the hydrotherapy phase first followed by observation; Group O (*n* = 2) completed observation first followed by the hydrotherapy phase.

A crossover design was used to evaluate hydrotherapy feasibility in males with MPSII (ISRCTN16919215). Using alternate allocation into two groups based on recruitment order, participants entered a 24‐week study consisting of two 12‐week phases. Group H started with hydrotherapy then moved to observation; Group O followed the reverse sequence (Figure 1). Blinding of the physiotherapist was unfeasible. However, to minimise bias, blinding was strictly maintained for the administrative research team during data entry from hard copies into Excel and for the statisticians throughout the analysis phase.

### 2.2 Recruitment and Consent

Potential participants were identified via the Salford Royal Hospital metabolic clinic and emailed information sheets at least 2 weeks before their appointments. Following screening and a protocol discussion with the clinical team, the Principal Investigator (PI) obtained written informed consent. Participants could withdraw at any time, though data collected up to that point was retained. The PI reserved the right to withdraw participants for medical reasons or adverse events, such as falls or excessive fatigue. Participants could also be withdrawn if they developed respiratory illness requiring antibiotics.

### 2.3 Eligibility Criteria

Eligible participants included males aged 16–58 with MPSII and persistent musculoskeletal pain, including those with reduced mobility. Inclusion required the ability to provide informed consent and passing a hydrotherapy risk assessment.

Exclusion criteria included standard hydrotherapy contraindications (e.g., acute gastrointestinal distress, recent cardiovascular events or exceeding evacuation weight limits), severe learning disabilities or total immobility. Those with LANSS scores indicating predominantly neuropathic pain were also excluded.

### 2.4 Intervention

Hydrotherapy was conducted in a specialist pool at Salford Royal Hospital, consisting of 12 weekly 25‐min sessions or 6 bi‐weekly 50‐min sessions. Access to the pool was facilitated via steps with staff assistance. Each session, led by a trained physiotherapist, comprised a warm‐up, upper/lower body exercises and a cool down tailored to each individual (see Supporting Information).

### 2.5 Data Collection

Outcome measures were collected at five timepoints: Baseline, Week 6, Week 12 (crossover point), Week 18 and Week 24 (study completion).

#### 2.5.1 Primary Outcome Measure (See Supporting Informations)

The primary endpoint was the change in health‐related quality of life (HRQoL), assessed via the Short Form Health Survey (SF‐36). The SF‐36 consists of 36 items, grouped into eight domains.

#### 2.5.2 Secondary Outcome Measures (See Supporting Informations)

Pain was assessed by the Brief Pain Inventory (BPI), which evaluates pain intensity and interference with daily activities. The BPI also collected data on current medications and the percentage of relief received from these treatments within the last 24 h. Reliance on pain control was evaluated using a medication questionnaire.

Physical functioning and mobility were assessed through the Timed Up and Go (TUAG), 6‐Minute Walk Test (6MWT) and the 10‐metre walk test (10MWT). Muscle strength was assessed by Quantitative Muscle Testing (QMT) with peak isometric contraction measured using handheld dynamometry (HHD). The testing was completed three times for each muscle group, with the max value recorded.

Psychological and emotional wellbeing were assessed using a battery of tools:

Anxiety was assessed using the Generalized Anxiety Disorder 7‐item (GAD‐7), depression severity by the Participant Health Questionnaire (PHQ‐9), fear of movement by the 17‐item Tampa Scale for Kinesiophobia (TSK‐17) and the participant's extent of catastrophic thinking towards pain using the Pain Catastrophizing Scale (PCS).

The Clinician Global Impression of Change (CGI‐C) was used to assess the participant's overall clinical improvement or worsening relative to the beginning of the study.

The Caregiver Global Impression of Severity (CaGIS) was used to capture the caregiver's perspective of the participant's current illness severity relative to other people in the same age bracket who do not have MPSII.

### 2.6 Statistical Analysis

Descriptive statistics are presented for the clinical outcomes; significance testing has not been undertaken. Data are presented as mean values at Week 0, Week 6, Week 12 and change from baseline to Week 12. Mean differences between the intervention and observation phases with confidence intervals (CI) are presented for some of the outcomes described in the text. Graphical representations of the data were created using Microsoft Excel.

## 3 Results

This study included five male participants with a confirmed diagnosis of MPSII, of which three were currently on ERT. The median age of the participants at the time of the study was 31 years, the mean age was 32.6 years (SD = 16.7) and the range was 18–59 years.

### 3.1 HRQoL

**FIGURE 2:**
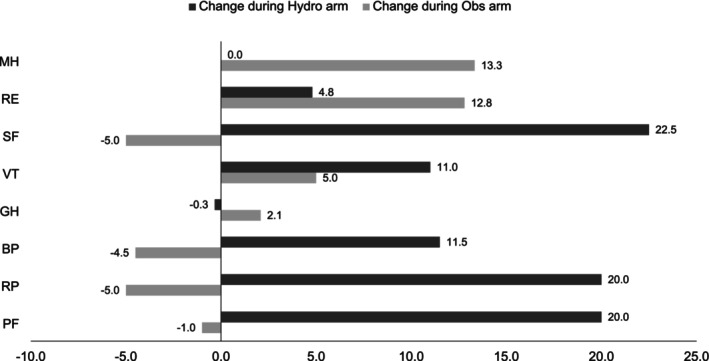
Graph showing the average change in SF‐36 domains regardless of group during the hydrotherapy arm (*n* = 5) and observation arm (*n* = 5). BP = bodily pain; GH = general health; MH = mental health; PF = Physical functioning; RE = role emotional; RP = role physical; SF = social functioning; SF‐36 = Short Form Health Survey 36‐item; VT = vitality.

**FIGURE 3:**
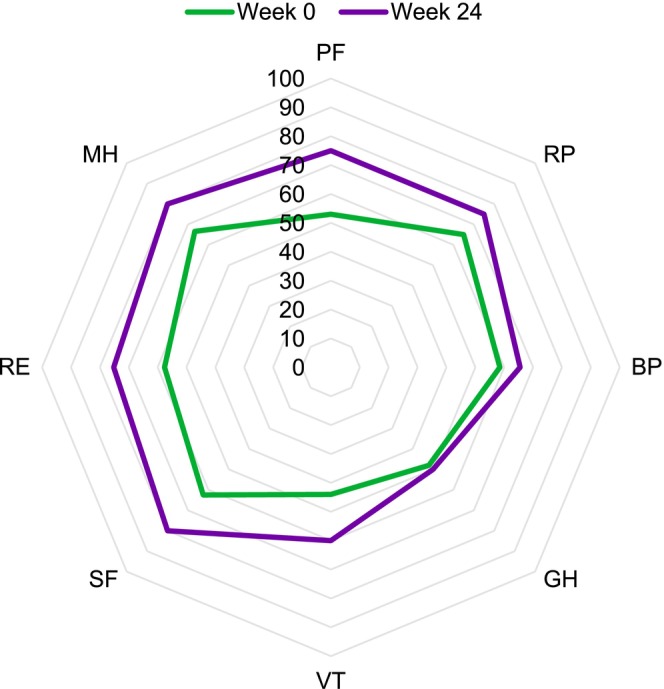
Radar chart comparing SF‐36 domain scores between Week 0 and Week 24. Each spoke on the radar chart represents an SF‐36 domain on a scale of 0–100, with higher scores representing better HRQoL. The domains are physical function (PF), role physical (RP), bodily pain (BP), general health (GH), vitality (VT), social function (SF), role emotional (RE) and mental health (MH). HRQoL = health‐related quality of life; SF‐36 = Short Form Health Survey 36‐item.

Health‐Related Quality of Life (HRQoL) was assessed using the SF‐36. Across all participants, mean score revealed patterns between the hydrotherapy and observation phases (Figures 2 and 3). During the hydrotherapy phase, participants demonstrated notable improvements across most physical and social domains, particularly in social functioning (SF), physical functioning (PF) and role physical (RP), which reported mean improvements of 22.5, 20.0 and 20.0, respectively. Interestingly, role emotional (RE) and mental health (MH) demonstrated their most substantial improvements during the observation phase rather than the hydrotherapy phase, with mean improvements of 12.8 and 13.3, respectively. Individual results for each SF‐36 domain are presented in Figure S1A–H.

### 3.2 Pain Severity and Interference With Daily Life

**FIGURE 4:**
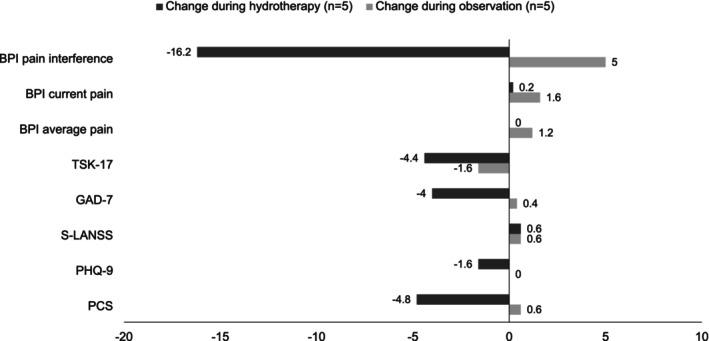
Graph showing the average change in pain and wellbeing scores regardless of group during the hydrotherapy arm (*n* = 5) and observation arm (*n* = 5). BPI = Brief Pain Inventory; GAD‐7 = Generalised Anxiety Disorder 7‐item; PCS = Pain Catastrophizing Scale; PHQ‐9 = Participant Health Questionnaire 9‐item; S‐LANSS = Self‐reported Leeds Assessment of Neuropathic Symptoms and Signs; TSK‐17 = Tampa Scale for Kinesiophobia 17‐item.

Average pain was measured via the Brief Pain Inventory (BPI; Q5), where higher scores indicate greater pain. Evaluation of these scores revealed that average pain remained stable during hydrotherapy (0 mean change) but increased by 1.2 during the observation phase, yielding a mean difference of −1.2 (95% CI −7.6 to 5.2; Figure 4). Current pain was similarly assessed using BPI Q6. Aggregate data showed only a minimal 0.2 mean increase in current pain during hydrotherapy, compared to a 1.6 mean increase during the observation arm. The most improvement across the study was recorded in pain interference (BPI Q9a–g). Combining all participants regardless of group sequence, those in the hydrotherapy period recorded a mean reduction of 16.2 (Figure 4). Individual BPI participant data are presented in and described in Figure S2A–C. The BPI recorded analgesic intake, including paracetamol, ibuprofen, co‐codamol and naproxen. Across the total cohort, the hydrotherapy arm reported 45.8% overall relief within 24 h—more than double the 18% reported by the observation arm.

### 3.3 Mental Health and Wellbeing

Anxiety, measured using the GAD‐7, improved across the cohort during the hydrotherapy arm with a mean change of −4.0 (Figure 4), whereas scores slightly worsened during the observation phase (+0.4), resulting in a mean difference of −5.6 (95% CI −10.4 to −0.8). Depression, assessed using the PHQ‐9, decreased by a mean of −1.6 during the hydrotherapy arm and did not change during the observation phase, resulting in a mean difference of −1.6 (95% CI −8.3 to 5.1; Figure 4).

Fear of movement was measured using the TSK‐17, where higher scores indicate a greater fear of pain, movement and injury. Globally, scores decreased by an average of −4.4 during the hydrotherapy arm compared to a −1.6 reduction during observation, yielding a mean difference of −2.8 (95% CI −16.8 to 11.2; Figure 4). Both groups demonstrated their greatest reductions in fear of movement during the active hydrotherapy intervention (Group H: −4.3; Group O: −4.5; Tables S1 and S2).

Pain catastrophising was assessed using the PCS, where lower scores indicate less catastrophising. On average, scores demonstrated a mean improvement of −4.8 during the hydrotherapy arm, whereas scores worsened by 0.6 during the observation phase (Figure 4). Observing sequence differences, Group H experienced the most substantial relief in catastrophising during their active hydrotherapy phase (−8.66; Table S1). Individual participant trajectories across the study arms are visualised and described in Figures S3–S7.

### 3.4 Mobility and Physical Functioning

**FIGURE 5:**
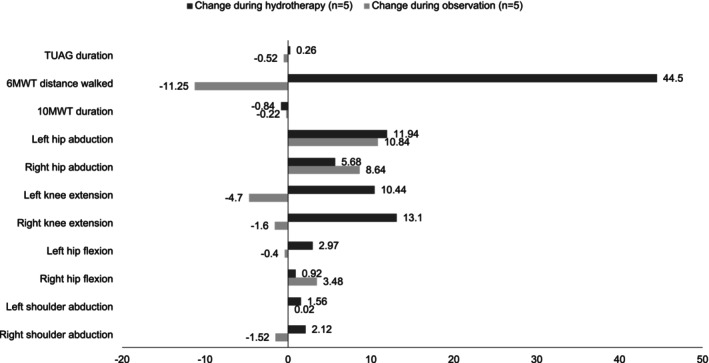
Graph showing the average change in mobility and muscle strength scores during the hydrotherapy arm (*n* = 5) and observation arm (*n* = 5). 6MWT = 6‐Minute Walk Test; 10MWT = 10‐metre walk test; TUAG: Timed Up and Go.

Hydrotherapy improved physical performance, specifically endurance and gait speed (Figure 5). The 6MWT showed the most substantial improvement across the study, with participants increasing their walking distance by a mean of 44.5 m during the hydrotherapy arm, compared to a mean reduction of 11.25 m during observation (Figure 5; mean difference 55.75, 95% CI −51.6 to 163.1). Gait speed, assessed via the 10MWT, similarly showed greater improvement during the hydrotherapy arm than during observation. Individual participant trajectories are visualised and described in Figures S8 and S9. Results for the TUAG test, where a quicker time indicates better functional mobility, were highly varied between the cohorts (Tables S1 and S2).

### 3.5 Muscle Strength

On average, participants achieved a mean strength increase of 6.1 lb. during the hydrotherapy arm, compared to a lesser gain of 1.85 lb. during the observation phase, with the greatest improvements specifically observed in hip abduction and knee extension strength (Figure 5).

### 3.6 Overall Clinical Improvement or Worsening

The CGI‐C assessed mobility, motor, daily living and psychosocial domains. During hydrotherapy, Participants 1 and 2 improved across all categories. Participants 3 and 4 remained largely stable until late‐stage improvements, whereas Participant 5 showed an immediate shift from ‘no clear change’ to ‘better’ across nearly all areas. In contrast, the observation phase showed a general lack of clinical change across the cohort.

### 3.7 Caregiver Assessment of Current Illness Severity

The CaGIS was used to evaluate the participants' current illness severity relative to age‐matched healthy peers, as perceived by their primary caregivers. Participant 1 showed the most dynamic profile: initially, walking and gross motor skills were rated ‘worse’ while mobility and daily living improved. By the study's end, gross motor skills stabilised to ‘no clear change’ and fine motor control improved. However, after the observation phase, fine motor and communication skills declined to ‘worse’, though other gains persisted. In contrast, Participant 5 showed consistent severity ratings across both phases, while Participants 2 and 3 remained stable regardless of the intervention. Data for Participant 4 were unavailable.

### 3.8 Untreated Versus ERT‐Treated Participants

Among untreated participants (*n* = 2), mean change scores revealed varied trajectories across the eight health domains (Table S3). During the hydrotherapy phase, the most prominent improvements in HRQoL occurred in social functioning (SF) and role emotional (RE), with mean increases of 18.8 (95% CI −60.7 to 98.2) and 16.0 (−187.3 to 219.3), respectively. Conversely, bodily pain was the sole domain to experience a mean decline. Functional capacity improved, evidenced by a mean increase of 62 (−166.7 to 290.7) metres in the 6MWT and a 6.4 (−19.7 to 32.4) lb. increase in average muscle strength. Participants reported decreased neuropathic pain, with S‐LANSS scores changing by an average of −5.0 (−68.5 to 58.5) (Figure S3) and increased kinesiophobia, with TSK scores rising by 2.0 (−61.5 to 65.5). Conversely, markers for anxiety (GAD‐7) and pain interference (BPI Q9 A‐G) showed mean improvements. In contrast, the observation arm showed a more static or slightly improved psychological state but worsening pain. During the observation phase, the majority of SF‐36 domain scores demonstrated a downward trend relative to the hydrotherapy phase. The most profound regression occurred in role‐physical (RP), with a mean change of −25.0 (−342.7 to 292.7) points. There were modest improvements in PHQ‐9 and TSK, yet pain scores deteriorated, with a 2.0 (−10.7 to 14.7) increase in immediate pain (BPI Q6) and a 2.5 (−130.9 to 135.9) increase in pain interference (BPI Q9 A‐G).

Among participants receiving ERT (*n* = 3), substantial improvements were observed across most SF‐36 physical domains during the hydrotherapy phase (Table S4). The most notable gains occurred in role‐physical (RP), physical functioning (PF), social functioning (SF) and bodily pain (BP), with mean improvements of 33.3 (−61.5 to 128.2), 30.0 (−39.2 to 99.2), 25.0 (−28.8 to 78.8) and 23.3 (−50.8 to 97.4), respectively. Participants demonstrated improvements in pain interference (BPI Q9 A‐G), pain catastrophizing (PCS) and kinesiophobia (TSK‐17). Functional capacity slightly improved, evidenced by a mean increase in the 6MWT and a 5.4 (−5.3 to 16.1) lb. increase in average muscle strength. The observation phase revealed a partial regression in several SF‐36 metrics, with social functioning (SF) and bodily pain (BP) scores decreasing by averages of 8.3 (−44.2 to 27.5) and 4.2 (−17.1 to 8.8) points, respectively. In contrast, mental health (MH) and role emotional (RE) scores vastly improved during this phase with mean improvements of 22.1 (−73.4 to 117.9) and 16.0 (−52.8 to 84.8), respectively. During the observation period, a moderate increase in BPI Q9 A‐G scores was reported, while muscle strength measurements remained relatively consistent with post‐intervention levels.

## 4 Discussion

This study demonstrates the feasibility of hydrotherapy as an adjunct to ERT for participants with MPSII. While ERT remains the gold standard for managing systemic GAG accumulation, restricted penetration of ERT into skeletal and joint tissues often leaves participants with persistent musculoskeletal morbidity [5, 8, 16].

Our results align with evidence that hydrostatic pressure in rehabilitation modulates pain and reduces joint stress, supported by findings in other rheumatic and musculoskeletal conditions [12, 17]. In our study, participants performed open and closed‐chain exercises that would otherwise be painful on land due to gravity‐based joint loading. These observations were similar to results found in populations with knee osteoarthritis [12]. Our data showed a mean 6MWT increase of 44.5 m, suggesting that hydrostatic unloading improves gait speed and endurance. Reductions in pain interference (mean −16.2), anxiety (GAD‐7) and kinesiophobia (TSK‐17) further highlight the intervention's holistic impact. These findings align with literature indicating that hydrotherapy reduces pain catastrophizing and improves confidence [18, 19, 20].

The benefits of hydrotherapy have been established in other rare conditions, such as Duchenne Muscular Dystrophy (DMD). In DMD, water is used to maintain muscle suppleness and independence without the risk of micro‐trauma associated with the lengthening phase of land‐based exercises [19]. In our study, MPSII participants achieved a mean strength increase of 6.1 lb. during the hydrotherapy arm without adverse effects. This corroborates the stance that water provides a safe medium for resistance training in fragile populations. Furthermore, while the Pediatric Outcomes Data Collection Instrument (PODCI) indicates that ERT improves global functioning, physical and pain domains often remain sub‐optimal [5]. Although these data must be interpreted with caution, our study revealed a divergence in outcomes between untreated participants and those receiving ERT during the hydrotherapy intervention. For untreated participants (*n* = 2), physical improvements in functional capacity and muscle strength were offset by an increase in kinesiophobia. The ERT cohort (*n* = 3) demonstrated gains in most SF‐36 physical domains and functional capacity, alongside reductions in pain catastrophising. This suggests that the benefits of hydrotherapy may be enhanced when paired with ERT (Tables S3 and S4).

Findings indicate that clinical changes were potentially driven by the active hydrotherapy phase. While all participants improved during hydrotherapy, the timing varied: Participant 5 responded immediately, whereas Participants 3 and 4 required several weeks of cumulative exposure. This suggests that while some may benefit instantly from the sensory properties of water, others need a longer intervention period for measurable improvements.

The data also highlights a potential disconnect between clinical assessment (CGI‐C) and caregiver perception (CaGIS), regarding the sustainability of these improvements. For Participant 1, the caregiver noted a perceived decline in fine motor and communication skills once hydrotherapy ended, with ratings regressing to ‘worse’ during the observation phase. This points to a ‘washout effect’, where the benefits of the intervention may diminish without continuous engagement. While our study did not include a formal washout period between phases, as is standard for non‐pharmacological interventions, this limits the interpretation of the crossover data. Consequently, potential carryover effects in either direction cannot be entirely excluded. Nonetheless, the data still reflect a natural regression once active treatment ceased. The improvements in environmental adaptation across multiple participants suggest that hydrotherapy provides holistic benefits that extend beyond physical mobility.

Due to the absence of prior studies on hydrotherapy in MPS, this study served as the first to specifically assess hydrotherapy in the MPSII population using a crossover design. Recruitment in rare disease research is inherently constrained by small patient populations and the necessity for strict inclusion criteria; notably, several interested participants were excluded due to comorbidities. Furthermore, logistical barriers such as travel distance to the centre also restricted participation. Given these constraints and the limited funding available, a target of five participants was agreed upon. While these preliminary results are encouraging, the inherent heterogeneity of the study cohort warrants a cautious interpretation and observed trends in overall data may be skewed by individual outliers.

## Author Contributions

**Andrew Oldham:** study design, data acquisition, data interpretation, clinical evaluation. **Mark Warburton**, **Nicole Stockton**, **Marie Meehan:** data acquisition. **Calvin Heal:** study design. **Karolina M. Stepien:** study concept, design, data analysis and review of first manuscript draft. All authors made substantial contributions to the interpretation of data for the work, revising the draft critically for important intellectual content and final approval of the version to be published.

## Funding

This work was supported by a grant from the Society for Mucopolysaccharide Diseases (Registered Charity No. 1143472. Registered as a Company limited by guarantee in England & Wales No. 7726882. Registered as a Charity in Scotland No. SCO41012).

## Ethics Statement

The study was conducted in accordance with the UK Policy Framework for Health and Social Care Research. The study received HRA approval from Greater Manchester South on 15 June 2023 (reference 23/NW/1069).

## Conflicts of Interest

The authors declare no conflicts of interest.

## Supporting information


**Figure S1A:** SF‐36 physical functioning (PF) domain. Higher scores denote a more favourable health state.
**Figure S1B:** SF‐36 role physical (RP) domain. Higher scores denote a more favourable health state.
**Figure S1C:** SF‐36 bodily pain (BP) domain. Higher scores denote a more favourable health state.
**Figure S1D:** SF‐36 general health (GH) domain. Higher scores denote a more favourable health state.
**Figure S1E:** SF‐36 vitality (VT) domain. Higher scores denote a more favourable health state.
**Figure S1F:** SF‐36 social functioning (SF) domain. Higher scores denote a more favourable health state.
**Figure S1G:** SF‐36 role emotional (RE) domain. Higher scores denote a more favourable health state.
**Figure S1H:** SF‐36 mental health (MH) domain. Higher scores denote a more favourable health state.
**Figure S2A:** Pain measured by the Brief Pain Inventory (BPI) Q5. The participants rated their ‘pain on average’ using a 0–10 Likert scale (where 0 is ‘no pain’ and 10 is ‘pain as bad as you can imagine’).
**Figure S2B:** Pain measured by the Brief Pain Inventory (BPI) Q6. The participants rated their ‘pain right now’ using a 0–10 Likert scale (where 0 is ‘no pain’ and 10 is ‘pain as bad as you can imagine’).
**Figure S2C:** Pain measured by the Brief Pain Inventory (BPI) Q9a‐g. Participants measured how much pain has interfered with general activity, mood, walking ability, normal work, relations with other people, sleep and enjoyment of life over the last 24 h using a 0–10 Likert scale (where 0 is ‘no pain’ and 10 is ‘pain as bad as you can imagine’).
**Figure S3:** Neuropathic pain assessed by the Self‐reported‐Leeds Assessment of Neuropathic Symptoms and Signs (S‐LANSS). The maximum score is 24 points and a score of 12 or higher suggests a likely neuropathic component to the patient's pain.
**Figure S4:** Anxiety assessed by the Generalised Anxiety Disorder 7‐item (GAD‐7). The severity of anxiety symptoms were measured over a 2‐week period by assigning a score from 0 to 21, with higher scores indicating severe anxiety.
**Figure S5:** Depression assessed by the Participant Health Questionnaire (PHQ‐9). Symptoms such as low mood, sleep disturbances and appetite changes are tracked over a 2‐week period and assigned a score from 0 to 27; higher totals indicate greater severity.
**Figure S6:** Fear of movement measured by the Tampa Scale of Kinesiophobia (TSK‐17). Patients answered 17 questions on a 4‐point Likert scale (1‐Strongly Disagree to 4‐Strongly Agree) yielding a total score. Higher scores indicate more fear and a score of ≥ 37 signifies clinical concern.
**Figure S7:** Catastrophic thinking towards pain was assessed using the Pain Catastrophizing Scale (PCS). Participants scored questions on a 5‐point Likert scale (0 = not at all to 4 = all the time), totalling 0–52. Higher scores indicated greater catastrophizing, with a total score above 30 representing a clinically significant level.
**Figure S8:** 6‐Minute Walk Test (6MWT). A sub‐maximal exercise test that measured the distance participants could walk on a flat, hard surface in 6 min.
**Figure S9:** Walking speed measured by the 10‐metre walk test (10MWT).
**Table S1:** Average scores for Group H (participants 1, 2 and 3).
**Table S2:** Average scores for Group O (participants 4 and 5).
**Table S3:** Average scores for untreated participants (participants 3 and 4).
**Table S4:** Average scores for participants receiving ERT (participants 1, 2 and 5).

## Data Availability

The data that supports the findings of this study are available in the S1 of this article.
