# Supplementary material for: The Impact of Hydrotherapy on Health‐Related Quality of Life, Pain and Mobility in Individuals With Mucopolysaccharidosis Type II (Hunter Syndrome): A Pilot Feasibility Study
Source: JIMD Rep. 2026 Aug 10;67(5):e70114. doi: 10.1002/jmd2.70114 (PMC13456980; doi:10.1002/jmd2.70114)
Supplement: Supplementary file 1 [file JMD2-67-e70114-s001.docx]

**Supplementary Materials**

**Methods**

*Intervention*

Core exercises included a circuit of resistance and range-of-motion movements including hip flexion and abduction, knee flexion, step-ups, squats, horizontal abduction, bicep curls, shoulder abduction, spinal rotations and bilateral shoulder flexion with a medicine ball. Spine mobilising and strengthening exercises for the spine and both upper and lower limbs were also performed.

*Primary outcome measure*

The Short Form Health Survey (SF-36) consists of 36 items (questions) that are grouped into eight domains which include physical functioning, social functioning, and mental health. SF-36 data was processed in two steps:

1. Recoding: Items were converted to a 0–100 scale, where higher scores indicate better health outcomes.

2. Aggregation: Items were grouped into 8 health scales by averaging the scores within each scale.

Scale scores were calculated based only on completed items; missing values were excluded from the average rather than imputed.

*Secondary outcome measures*

Pain was assessed by the Brief Pain Inventory (BPI). The participants rated their “pain on average” and “pain right now” using a 0–10 Likert scale (where 0 is "no pain" and 10 is "pain as bad as you can imagine"). Pain interference measured how much pain has interfered with general activity, mood, walking ability, normal work, relations with other people, sleep and enjoyment of life over the last 24 hours (also using a 0–10 Likert scale).

Anxiety was assessed using the Generalized Anxiety Disorder 7-item (GAD-7). This seven question screening tool measures the severity of anxiety symptoms over a two-week period by assigning a score from 0 to 21, with higher scores indicating severe anxiety.

Depression severity was assessed using the Participant Health Questionnaire (PHQ-9), a validated nine-item screening tool. By tracking symptoms such as low mood, sleep disturbances, and appetite changes over a two-week period, the PHQ-9 assigns a score from 0 to 27; higher totals indicate greater severity, allowing clinicians to categorize the depression from minimal to severe.

Fear of movement was assessed using the 17-item Tampa Scale for Kinesiophobia (TSK-17), which evaluated the association between physical activity and the risk of reinjury. Participants rated 17 statements on a 4-point Likert scale (1 to 4), yielding a total score of 17–68, where higher values indicate greater levels of kinesiophobia.

The Pain Catastrophizing Scale (PCS), a 13-item instrument, was used to quantify a participant’s extent of catastrophic thinking toward pain across three dimensions: rumination, magnification, and helplessness. Participants rated their thoughts and feelings on a 5-point Likert scale (0 to 4), resulting in a total score of 0–52, where higher values indicated a clinically significant level of catastrophising.

The Clinician Global Impression of Change (CGI-C) was used to assess the participant’s overall clinical improvement or worsening relative to the beginning of the study. The consultant, physiotherapist or nurse rated the change on a 5-point Likert scale, ranging from 1 (much worse) to 5 (much better). The scale includes 5 grades: much worse, worse, no clear change, better, much better. It is an observer-rated tool used to measure how much a patient's condition has changed relative to their baseline. There is no mathematical formula required to calculate the change; instead, the clinician directly scores the change from memory or prior notes.

The Caregiver Global Impression of Severity (CaGIS) was used to capture the caregiver’s perspective of the participant’s current illness severity relative to other people in the same age bracket who do not have MPSII. The caregiver rated the participant’s overall condition on a 5-point Likert scale, ranging from 1 (very much worse than others) to 5 (no clear difference from others).

**Statistical analysis:**

Confidence intervals were calculated as for a paired t-test: mean difference ± t multiplied by the standard error, where t is the critical t-value with n−1 degrees of freedom

**Results: Individual participant trajectories**

*HRQoL*

Individual trajectories for physical functioning (PF) varied slightly following the cessation of hydrotherapy (Suppl Figure 1A). A slight increase in physical functioning (PF) scores at the end of the hydrotherapy phase relative to the observation phase were reported by Participant 1 (100 vs. 95) and Participant 4 (60 vs. 55). physical functioning (PF) scores remained unchanged between the hydrotherapy and observation phases for Participant 3 (80 vs. 80) and Participant 5 (40 vs. 40). Participant 2 demonstrated a slight decrease in physical functioning (PF) score at the end of the hydrotherapy phase relative to the observation phase (80 vs. 85).

Most participants experienced no change in their role physical (RP) scores between the two phases (Suppl Figure 1B). Participants 1, 3, and 5 maintained maximum scores of 100, while Participant 4 reported a score of 0 at the end of both phases. Participant 2 demonstrated a notable decrease in role physical (RP) score at the end of the hydrotherapy phase relative to the observation phase (75 vs. 100).

Individual trajectories for bodily pain (BP) scores varied following the cessation of hydrotherapy (Suppl Figure 1C). A slight increase in bodily pain (BP) scores at the end of the hydrotherapy phase relative to the observation phase were reported by Participant 1 (100 vs. 90), Participant 2 (80 vs. 77.5), and Participant 4 (67.5 vs. 57.5), indicating an increase in pain during the observation phase. Bodily pain (BP) scores remained unchanged between the hydrotherapy and observation phases for Participant 3 (45 vs. 45). Participant 5 demonstrated a notable decrease in bodily pain (BP) score at the end of the hydrotherapy phase relative to the observation phase (47.5 vs. 57.5).

A slight decrease in general health (GH) scores at the end of the hydrotherapy phase relative to the observation phase were reported by Participant 1 (80 vs. 90), Participant 2 (30 vs. 35), and Participant 3 (45 vs. 50) (Suppl Figure 1D). General health (GH) scores remained unchanged between the hydrotherapy and observation phases for Participant 5 (40 vs. 40). Participant 4 demonstrated a slight increase in general health (GH) score at the end of the hydrotherapy phase relative to the observation phase (38.5 vs. 35.1).

Most participants demonstrated a decrease in vitality (VT) scores at the end of the hydrotherapy phase relative to the observation phase (Suppl Figure 1E). This trend was reported by Participant 1 (75 vs. 85), Participant 2 (55 vs. 60), Participant 3 (60 vs. 65), and Participant 5 (75 vs. 80). Vitality (VT) scores remained unchanged between the hydrotherapy and observation phases for Participant 4 (15 vs. 15).

Individual trajectories for social functioning (SF) scores varied following the end of hydrotherapy (Suppl Figure 1F). A slight increase in social functioning (SF) scores at the end of the hydrotherapy phase relative to the observation phase were reported by Participant 1 (100 vs. 75) and Participant 4 (62.5 vs. 50). Social functioning (SF) scores remained unchanged between the hydrotherapy and observation phases for Participant 2 (62.5 vs. 62.5), Participant 3 (100 vs. 100), and Participant 5 (100 vs. 100).

Most participants experienced no change in their role emotional (RE) scores between the two phases (Suppl Figure 1G). Participants 2 (56 vs. 56), 4 (48 vs. 48), and 5 (92 vs. 92) maintained stable scores at the end of both phases. In contrast, a decrease in role emotional (RE) scores at the end of the hydrotherapy phase relative to the observation phase were reported by Participant 1 (40 vs. 88) and Participant 3 (84 vs. 92).

Most participants experienced no change in their mental health (MH) scores between the two phases (Suppl Figure 1H). Participants 1, 3, and 5 maintained maximum scores of 100, while Participant 4 reported a score of 0 at the end of both phases. Participant 2 demonstrated a notable decrease in mental health (MH) score at the end of the hydrotherapy phase relative to the observation phase (33.3 vs. 100).

*Pain severity and interference with daily life*

Average pain was measured via the Brief Pain Inventory (BPI; Q5), where higher scores indicate greater pain. Mixed individual responses at the end of the hydrotherapy phase were reported; lower pain scores relative to the observation phase were reported by Participant 1 (4 vs. 6), Participant 2 (0 vs. 4), and Participant 4 (1 vs. 3), whereas Participant 3 (6 vs. 5) and Participant 5 (7 vs. 5) demonstrated slight increases in pain following the intervention.

Current pain was similarly assessed using BPI Q6. Individually, lower pain scores at the end of the hydrotherapy phase relative to the observation phase were reported by Participant 2 (0 vs. 5), Participant 3 (3 vs. 4), and Participant 4 (2 vs. 4). Conversely, scores remained unchanged between the hydrotherapy and observation phases for Participant 1 (4 vs. 4) and Participant 5 (7 vs. 7).

Pain interference was measured using BPI Q9a–g. At the individual level, this decline was driven by improvements at the end of the hydrotherapy phase relative to the observation phase for Participant 4 (1 vs. 21) and Participant 5 (12 vs. 40). In contrast, Participant 1 (5 vs. 2) and Participant 3 (30 vs. 22) demonstrated increases in pain interference following the intervention and the score remained unchanged for Participant 2 (0 vs. 0).

*Mental health and wellbeing*

Anxiety was measured using the GAD-7. At the individual level, lower anxiety scores at the end of the hydrotherapy phase relative to the observation phase were reported by Participant 1 (1 vs. 2), Participant 2 (2 vs. 6), and Participant 4 (6 vs. 9). Conversely, scores remained unchanged between phases for Participant 3 (1 vs. 1) and Participant 5 (0 vs. 0).

Depression was assessed using the PHQ-9. At the individual level, lower depression scores at the end of the hydrotherapy phase relative to the observation phase were reported by Participant 1 (0 vs. 2) and Participant 5 (0 vs. 1). Meanwhile, scores increased slightly for Participant 2 (2 vs. 1), Participant 3 (3 vs. 1) and Participant 4 (16 vs. 15).

Fear of movement was measured using the TSK-17. Individual responses at the end of the hydrotherapy phase were highly varied. Lower scores (indicating improvement) relative to the observation phase were reported by Participant 4 (40 vs. 43) and Participant 5 (22 vs. 28). Conversely, higher scores relative to the observation phase were demonstrated by Participant 1 (30 vs. 28), Participant 2 (37 vs. 36), and Participant 3 (32 vs. 27).

Pain catastrophising was assessed using the PCS. At the individual level, the positive trend during hydrotherapy was primarily driven by Participant 1, who reported a lower score relative to the observation phase (1 vs. 5). In contrast, Participant 2 (2 vs. 1) and Participant 4 (37 vs. 35) reported slightly increased scores at the end of the hydrotherapy phase. Scores remained unchanged between phases for Participant 3 (1 vs. 1) and Participant 5 (0 vs. 0).

*Mobility and physical functioning*

For the 6MWT at the individual level, a greater distance walked at the end of the hydrotherapy phase relative to the observation phase was reported by Participant 1 (580 vs. 556 m) and Participant 4 (456 vs. 412 m). Conversely, Participant 2 (407 vs. 414 m) and Participant 3 (660 vs. 666 m) reported slightly less distance walked at the end of hydrotherapy, while Participant 5 did not complete the 6MWT assessment.

Gait speed was assessed via the 10MWT. A positive trend was reflected at the individual level, where a quicker time relative to the observation phase was reported by Participant 2 (5.2 vs. 5.3 s), Participant 4 (6.8 vs. 7.7 s), and Participant 5 (9.7 vs. 11.1 s). Conversely, times remained unchanged between phases for Participant 1 (5 vs. 5 s) and Participant 3 (4.6 vs. 4.6 s).

The TUAG test measured functional mobility. The majority of participants recorded a slower time at the end of the hydrotherapy phase relative to the observation phase: Participant 1 (5.4 vs. 5.2 s), Participant 2 (5 vs. 4.8 s), Participant 4 (7.2 vs. 6.1 s), and Participant 5 (8.7 vs. 8.3 s). Participant 3 (5.6 vs. 5.6 s) remained unchanged between phases.

*Muscle strength*

Substantial individual gains at the end of the hydrotherapy phase relative to the observation phase were reported for Participant 4 (46.7 vs. 38.3 lb) and Participant 5 (34.2 vs. 27.5 lb). Conversely, the remaining cohort demonstrated declines in average muscle strength following the intervention, as seen in Participant 1 (58.8 vs. 59.1 lb), Participant 2 (42.7 vs. 48.4 lb), and Participant 3 (61.8 vs. 65.9 lb).

|  | Participant 1 | Participant 2 | Participant 3 | Participant 4 | Participant 5 |
| --- | --- | --- | --- | --- | --- |
| Hydro 1 | 65 | 25 | 75 |  |  |
| Hydro 2 | 55 | 90 | 85 |  |  |
| Hydro 3/Obs 1 | 100 | 80 | 80 | 55 | 45 |
| Obs 2 | 90 | 50 | 85 | 60 | 70 |
| Obs 3/ Hydro 1 | 95 | 85 | 80 | 55 | 40 |
| Hydro 2 |  |  |  | 60 | 45 |
| Hydro 3 |  |  |  | 60 | 40 |

**Supplementary Figure 1A. SF-36 physical functioning (PF) domain.** Higher scores denote a more favourable health state.

|  | Participant 1 | Participant 2 | Participant 3 | Participant 4 | Participant 5 |
| --- | --- | --- | --- | --- | --- |
| Hydro 1 | 75 | 0 | 99.5 |  |  |
| Hydro 2 | 50 | 100 | 99.5 |  |  |
| Hydro 3/Obs 1 | 100 | 75 | 99.5 | 50 | 100.5 |
| Obs 2 | 50 | 25 | 99.5 | 75 | 100.5 |
| Obs 3/ Hydro 1 | 100 | 100 | 99.5 | 0 | 100.5 |
| Hydro 2 |  |  |  | 100 | 100.5 |
| Hydro 3 |  |  |  | 0 | 100.5 |

**Supplementary Figure 1B. SF-36 role physical (RP) domain.** Higher scores denote a more favourable health state.

|  | Participant 1 | Participant 2 | Participant 3 | Participant 4 | Participant 5 |
| --- | --- | --- | --- | --- | --- |
| Hydro 1 | 67.5 | 32.5 | 67.5 |  |  |
| Hydro 2 | 80 | 80 | 90 |  |  |
| Hydro 3/Obs 1 | 100 | 80 | 45 | 67.5 | 57.5 |
| Obs 2 | 90 | 45 | 57.5 | 67.5 | 47.5 |
| Obs 3/ Hydro 1 | 90 | 77.5 | 45 | 57.5 | 57.5 |
| Hydro 2 |  |  |  | 57.5 | 22.5 |
| Hydro 3 |  |  |  | 67.5 | 47.5 |

**Supplementary Figure 1C. SF-36 bodily pain (BP) domain.** Higher scores denote a more favourable health state.

|  | Participant 1 | Participant 2 | Participant 3 | Participant 4 | Participant 5 |
| --- | --- | --- | --- | --- | --- |
| Hydro 1 | 55 | 60 | 45 |  |  |
| Hydro 2 | 70 | 68.75 | 60 |  |  |
| Hydro 3/Obs 1 | 80 | 30 | 45 | 39.7222 | 45 |
| Obs 2 | 75 | 40 | 45 | 43.8889 | 55 |
| Obs 3/ Hydro 1 | 90 | 35 | 50 | 35.1389 | 40 |
| Hydro 2 |  |  |  | 46.8056 | 45 |
| Hydro 3 |  |  |  | 38.4722 | 40 |

**Supplementary Figure 1D. SF-36 general health (GH) domain.** Higher scores denote a more favourable health state.

|  | Participant 1 | Participant 2 | Participant 3 | Participant 4 | Participant 5 |
| --- | --- | --- | --- | --- | --- |
| Hydro 1 | 45 | 45 | 40 |  |  |
| Hydro 2 | 45 | 50 | 80 |  |  |
| Hydro 3/Obs 1 | 75 | 55 | 60 | 5 | 85 |
| Obs 2 | 75 | 60 | 70 | 15 | 90 |
| Obs 3/ Hydro 1 | 85 | 60 | 65 | 15 | 80 |
| Hydro 2 |  |  |  | 10 | 85 |
| Hydro 3 |  |  |  | 15 | 75 |

**Supplementary Figure 1E. SF-36 vitality (VT) domain.** Higher scores denote a more favourable health state.

|  | Participant 1 | Participant 2 | Participant 3 | Participant 4 | Participant 5 |
| --- | --- | --- | --- | --- | --- |
| Hydro 1 | 62.5 | 25 | 75 |  |  |
| Hydro 2 | 62.5 | 87.5 | 99.5 |  |  |
| Hydro 3/Obs 1 | 100 | 62.5 | 99.5 | 50 | 100.5 |
| Obs 2 | 62.5 | 50 | 99.5 | 62.5 | 100.5 |
| Obs 3/ Hydro 1 | 75 | 62.5 | 99.5 | 50 | 100.5 |
| Hydro 2 |  |  |  | 50 | 100.5 |
| Hydro 3 |  |  |  | 62.5 | 100.5 |

**Supplementary Figure 1F. SF-36 social functioning (SF) domain.** Higher scores denote a more favourable health state.

|  | Participant 1 | Participant 2 | Participant 3 | Participant 4 | Participant 5 |
| --- | --- | --- | --- | --- | --- |
| Hydro 1 | 60 | 44 | 52 |  |  |
| Hydro 2 | 48 | 56 | 92 |  |  |
| Hydro 3/Obs 1 | 39.5 | 56 | 84 | 40.5 | 92 |
| Obs 2 | 31.5 | 56 | 88 | 32.5 | 100 |
| Obs 3/ Hydro 1 | 88 | 56 | 92 | 48 | 92 |
| Hydro 2 |  |  |  | 36 | 96 |
| Hydro 3 |  |  |  | 48 | 92 |

**Supplementary Figure 1G. SF-36 role emotional (RE) domain.** Higher scores denote a more favourable health state.

|  | Participant 1 | Participant 2 | Participant 3 | Participant 4 | Participant 5 |
| --- | --- | --- | --- | --- | --- |
| Hydro 1 | 100 | 33.3333 | 101 |  |  |
| Hydro 2 | 33.3 | 100 | 101 |  |  |
| Hydro 3/Obs 1 | 99 | 33.3333 | 101 | 0 | 100 |
| Obs 2 | 99 | 100 | 101 | 0 | 100 |
| Obs 3/ Hydro 1 | 99 | 100 | 101 | 0 | 100 |
| Hydro 2 |  |  |  | 0 | 100 |
| Hydro 3 |  |  |  | 0 | 100 |

**Supplementary Figure 1H. SF-36 mental health (MH) domain.** Higher scores denote a more favourable health state.

|  | Participant 1 | Participant 2 | Participant 3 | Participant 4 | Participant 5 |
| --- | --- | --- | --- | --- | --- |
| Hydro 1 | 0 | 5 | 5 |  |  |
| Hydro 2 | 3 | 5 | 6 |  |  |
| Hydro 3/Obs 1 | 4 | 0 | 6 | 1 | 6 |
| Obs 2 | 6 | 8 | 7 | 2 | 8 |
| Obs 3/ Hydro 1 | 6 | 4 | 5 | 3 | 5 |
| Hydro 2 |  |  |  | 3 | 5 |
| Hydro 3 |  |  |  | 1 | 7 |

**Supplementary Figure 2A. Pain measured by the Brief Pain Inventory (BPI) Q5.** The participants rated their “pain on average” using a 0–10 Likert scale (where 0 is "no pain" and 10 is "pain as bad as you can imagine").

|  | Participant 1 | Participant 2 | Participant 3 | Participant 4 | Participant 5 |
| --- | --- | --- | --- | --- | --- |
| Hydro 1 | 0 | 0 | 4 |  |  |
| Hydro 2 | 4 | 5 | 5 |  |  |
| Hydro 3/Obs 1 | 4 | 0 | 3 | 1 | 8 |
| Obs 2 | 3 | 8 | 3 | 2 | 5 |
| Obs 3/ Hydro 1 | 4 | 5 | 4 | 4 | 7 |
| Hydro 2 |  |  |  | 3 | 6 |
| Hydro 3 |  |  |  | 2 | 7 |

**Supplementary Figure 2B. Pain measured by the Brief Pain Inventory (BPI) Q6.** The participants rated their “pain right now” using a 0–10 Likert scale (where 0 is "no pain" and 10 is "pain as bad as you can imagine").

|  | Participant 1 | Participant 2 | Participant 3 | Participant 4 | Participant 5 |
| --- | --- | --- | --- | --- | --- |
| Hydro 1 | 29 | 23 | 16 |  |  |
| Hydro 2 | 29 | 2 | 28 |  |  |
| Hydro 3/Obs 1 | 5 | 0 | 30 | 8 | 17 |
| Obs 2 | 13 | 24 | 15 | 9 | 21 |
| Obs 3/ Hydro 1 | 2 | 0 | 22 | 21 | 40 |
| Hydro 2 |  |  |  | 12 | 13 |
| Hydro 3 |  |  |  | 1 | 12 |

**Supplementary Figure 2C. Pain measured by the Brief Pain Inventory (BPI) Q9a-g.** Participants measured how much pain has interfered with general activity, mood, walking ability, normal work, relations with other people, sleep and enjoyment of life over the last 24 hours using a 0–10 Likert scale (where 0 is "no pain" and 10 is "pain as bad as you can imagine").

|  | Participant 1 | Participant 2 | Participant 3 | Participant 4 | Participant 5 |
| --- | --- | --- | --- | --- | --- |
| Hydro 1 | 0.2 | 2 | 10 |  |  |
| Hydro 2 | 0.1 | -0.1 | 0 |  |  |
| Hydro 3/Obs 1 | 0.1 | -0.1 | 0 | 0 | 16 |
| Obs 2 | 0.1 | -0.1 | 0 | 5 | 8 |
| Obs 3/ Hydro 1 | 5 | -0.1 | 0 | 0 | 8 |
| Hydro 2 |  |  |  | 0 | 13 |
| Hydro 3 |  |  |  | 3 | 8 |

**Supplementary Figure 3. Neuropathic pain assessed by the Self-reported- Leeds Assessment of Neuropathic Symptoms and Signs (S-LANSS).** The maximum score is 24 points and a score of 12 or higher suggests a likely neuropathic component to the patient's pain.

|  | Participant 1 | Participant 2 | Participant 3 | Participant 4 | Participant 5 |
| --- | --- | --- | --- | --- | --- |
| Hydro 1 | 4 | 6 | 11 |  |  |
| Hydro 2 | 4 | 6 | 2 |  |  |
| Hydro 3/Obs 1 | 1 | 2 | 1 | 12 | 0 |
| Obs 2 | 0 | 1 | 1 | 6 | 2 |
| Obs 3/ Hydro 1 | 2 | 6 | 1 | 9 | 0 |
| Hydro 2 |  |  |  | 9 | 1 |
| Hydro 3 |  |  |  | 6 | 0 |

**Supplementary Figure 4. Anxiety assessed by the Generalized Anxiety Disorder 7-item (GAD-7).** The severity of anxiety symptoms were measured over a two-week period by assigning a score from 0 to 21, with higher scores indicating severe anxiety.

|  | Participant 1 | Participant 2 | Participant 3 | Participant 4 | Participant 5 |
| --- | --- | --- | --- | --- | --- |
| Hydro 1 | 8 | 8 | 9 |  |  |
| Hydro 2 | 5 | 6 | 1 |  |  |
| Hydro 3/Obs 1 | 0 | 2 | 3 | 19 | 0 |
| Obs 2 | 6 | 0 | 2 | 16 | 0 |
| Obs 3/ Hydro 1 | 2 | 1 | 1 | 15 | 1 |
| Hydro 2 |  |  |  | 15 | 0 |
| Hydro 3 |  |  |  | 16 | 0 |

**Supplementary Figure 5. Depression assessed by the Participant Health Questionnaire (PHQ-9).** Symptoms such as low mood, sleep disturbances, and appetite changes are tracked over a two-week period and assigned a score from 0 to 27; higher totals indicate greater severity.

|  | Participant 1 | Participant 2 | Participant 3 | Participant 4 | Participant 5 |
| --- | --- | --- | --- | --- | --- |
| Hydro 1 | 51 | 36 | 25 |  |  |
| Hydro 2 | 37 | 45 | 36 |  |  |
| Hydro 3/Obs 1 | 30 | 37 | 32 | 42 | 29 |
| Obs 2 | 30 | 36 | 34 | 38 | 20 |
| Obs 3/ Hydro 1 | 28 | 36 | 27 | 43 | 28 |
| Hydro 2 |  |  |  | 47 | 26 |
| Hydro 3 |  |  |  | 40 | 22 |

**Supplementary Figure 6. Fear of movement measured by the Tampa Scale of Kinesiophobia (TSK-17).** Patients answered 17 questions on a 4-point Likert scale (1-Strongly Disagree to 4-Strongly Agree) yielding a total score. Higher scores indicate more fear and a score of ≥37 signifies clinical concern.

|  | Participant 1 | Participant 2 | Participant 3 | Participant 4 | Participant 5 |
| --- | --- | --- | --- | --- | --- |
| Hydro 1 | 18 | 10 | 2 |  |  |
| Hydro 2 | 22 | 0 | 0 |  |  |
| Hydro 3/Obs 1 | 1 | 2 | 1 | 34 | 1 |
| Obs 2 | 0 | 9 | 1 | 37 | 0 |
| Obs 3/ Hydro 1 | 5 | 1 | 1 | 35 | 0 |
| Hydro 2 |  |  |  | 39 | 0 |
| Hydro 3 |  |  |  | 37 | 0 |

**Supplementary Figure 7. Catastrophic thinking toward pain was assessed using the Pain Catastrophizing Scale (PCS).** Participants scored questions on a 5-point Likert scale (0=not at all to 4=all the time), totalling 0–52. Higher scores indicated greater catastrophizing, with a total score above 30 representing a clinically significant level.

|  | Participant 1 | Participant 2 | Participant 3 | Participant 4 |
| --- | --- | --- | --- | --- |
| Hydro 1 | 491 | 442 | 580 |  |
| Hydro 2 | 563 | 0 | 668 |  |
| Hydro 3/Obs 1 | 580 | 407 | 660 | 446 |
| Obs 2 | 554 | 170 | 651 | 433 |
| Obs 3/ Hydro 1 | 556 | 414 | 666 | 412 |
| Hydro 2 |  |  |  | 455 |
| Hydro 3 |  |  |  | 456 |

**Supplementary Figure 8. 6-Minute Walk Test (6MWT).** A sub-maximal exercise test that measured the distance participants could walk on a flat, hard surface in 6 minutes.

|  | Participant 1 | Participant 2 | Participant 3 | Participant 4 | Participant 5 |
| --- | --- | --- | --- | --- | --- |
| Hydro 1 | 6.6 | 4.8 | 5.3 |  |  |
| Hydro 2 | 4.5 | 4.9 | 4.7 |  |  |
| Hydro 3/Obs 1 | 5 | 5.2 | 4.6 | 7.8 | 10 |
| Obs 2 | 5.1 | 5.2 | 4.9 | 7.3 | 9.8 |
| Obs 3/ Hydro 1 | 5 | 5.3 | 4.6 | 7.7 | 11.1 |
| Hydro 2 |  |  |  | 7.1 | 10 |
| Hydro 3 |  |  |  | 6.8 | 9.7 |

**Supplementary Figure 9. Walking speed measured by the 10-metre walk test (10MWT).**

**Supplementary Table 1. Average scores for Group H (participants 1,2 and 3).**

|  | **Group H**  **Hydrotherapy** | | | | **Group H**  **Observation** | | | |
| --- | --- | --- | --- | --- | --- | --- | --- | --- |
|  | **Week 0** | **Week 6** | **Week 12** | **Change over  Week 0–12** | **Week 12** | **Week 18** | **Week 24** | **Change over Week 12–24** |
| **HRQoL, pain and wellbeing** |  |  |  |  |  |  |  |  |
| PCS | 10.0 | 7.3 | 1.3 | -8.7 | 1.3 | 3.3 | 2.3 | 1.0 |
| PHQ 9 | 2.6 | 2.0 | 0.0 | -2.7 | 0.0 | 2.0 | 1.0 | 1.0 |
| S-LANSS | 20.0 | 24.0 | 24.0 | 4.0 | 24.0 | 24.0 | 22.3 | -1.7 |
| GAD 7 | 7.0 | 4.0 | 1.3 | -5.7 | 1.3 | 0.6 | 3.0 | 1.7 |
| TSK | 37.3 | 39.3 | 33.0 | -4.3 | 33.0 | 33.3 | 30.3 | -2.7 |
| BPI |  |  |  |  |  |  |  |  |
| Q5 Pain | 3.3 | 4.7 | 3.3 | 0.0 | 3.3 | 7.0 | 5.0 | 1.7 |
| Q6 Pain felt right now | 1.3 | 4.7 | 2.3 | 1.0 | 2.3 | 4.7 | 4.3 | 2.0 |
| Q9 A-G^a^ | 3.2 | 2.8 | 1.7 | -1.6 | 1.7 | 2.5 | 1.1 | -0.5 |
| SF36 |  |  |  |  |  |  |  |  |
| PF | 55.0 | 76.7 | 86.7 | 31.7 | 86.7 | 75.0 | 86.7 | 0.0 |
| RP | 58.3 | 83.3 | 91.7 | 33.3 | 91.7 | 58.3 | 100.0 | 8.3 |
| BP | 55.8 | 83.3 | 75.0 | 19.2 | 75.0 | 64.2 | 70.8 | -4.2 |
| GH | 53.3 | 66.3 | 51.7 | -1.7 | 51.7 | 53.3 | 58.3 | 6.7 |
| VT | 43.3 | 58.3 | 63.3 | 20.0 | 63.3 | 68.3 | 70.0 | 6.7 |
| SF | 54.2 | 83.3 | 87.5 | 33.3 | 87.5 | 70.8 | 79.2 | -8.3 |
| RE | 52.0 | 65.3 | 60.0 | 8.0 | 60.0 | 58.7 | 78.7 | 18.7 |
| MH | 77.8 | 77.8 | 77.8 | 0.0 | 77.8 | 100.0 | 100.0 | 22.2 |
| **Mobility** | 55.0 | 76.7 | 86.7 | 31.7 | 86.7 | 75.0 | 86.7 | 0.0 |
| 10MWT (sec) | 5.6 | 4.7 | 4.9 | -0.6 | 4.9 | * | 5.0 | 0.0 |
| 6MWT (m) | 504.0 | * | 549.0 | 45.0 | 549.0 | 458.3 | 545.3 | -4.7 |
| TUAG (sec) | 5.4 | 5.1 | 5.3 | -0.1 | 5.3 | * | 5.2 | -1.3 |
| **Muscle strength (lb)** |  |  |  |  |  |  |  |  |
| Right Shoulder Abduction | 48.8 | 41.9 | 51.9 | 3.1 | 51.9 | * | 50.1 | -1.9 |
| Left Shoulder Abduction | 47.8 | 40.4 | 49.6 | 1.8 | 49.6 | * | 48.5 | -1.1 |
| Right Hip Flexion^b^ | 34.1 | 32.1 | 37.3 | 3.2 | 37.3 | 35.8 | 38.9 | 1.7 |
| Left Hip Flexion^b^ | 36.5 | 35.0 | 41.4 | 4.9 | 41.4 | 38.3 | 40.1 | -1.3 |
| Right Knee Extension | 63.8 | 64.8 | 80.7 | 16.9 | 80.7 | * | 78.4 | -2.3 |
| Left Knee Extension | 70.3 | 58.4 | 74.0 | 3.7 | 74.0 | * | 66.6 | -7.4 |
| Right Hip Abduction | 50.5 | 44.2 | 50.5 | 0.0 | 50.5 | * | 67.6 | 17.1 |
| Left Hip Abduction | 38.9 | 38.7 | 48.0 | 9.1 | 48.0 | * | 66.3 | 18.3 |
| Average muscle strength | 48.8 | 44.4 | 54.2 | 5.3 | 54.2 | * | 57.0 | 2.9 |

^a^ Pain interference measured how much pain has interfered with general activity, mood, walking ability, normal work, relations with other people, sleep and enjoyment of life over the last 24 hours.

^b^ Participants assessed n=2.

*Indicates missing data.

*Abbreviations: 6MWT= 6-Minute Walk Test; 10MWT= 10-metre walk test; BPI= Brief Pain Inventory; GAD-7= Generalized Anxiety Disorder 7-item; HRQoL= health-related quality of life; PCS= Pain Catastrophizing Scale; PHQ-9= Participant Health Questionnaire 9-item; S-LANSS= Self-reported- Leeds Assessment of Neuropathic Symptoms and Signs; SF-36= Short Form Health Survey 36-item; TSK-17= Tampa Scale for Kinesiophobia 17-item; TUAG: Timed Up and Go.*

**Supplementary Table 2. Average scores for Group O (participants 4 and 5).**

|  | **Group O**  **Observation** | | | | **Group O**  **Hydrotherapy** | | | |
| --- | --- | --- | --- | --- | --- | --- | --- | --- |
|  | **Week 0** | **Week 6** | **Week 12** | **Change over  Week 0–12** | **Week 12** | **Week 18** | **Week 24** | **Change over Week 12–24** |
| **HRQoL, pain and wellbeing** |  |  |  |  |  |  |  |  |
| PCS | 24.0 | 24.5 | 24.0 | 0.0 | 24.0 | 26.0 | 18.5 | -5.5 |
| PHQ 9 | 0.0 | 0.0 | 1.0 | 1.0 | 1.0 | 0.0 | 8.0 | 7.0 |
| S-LANSS | 8.0 | 16.0 | 16.0 | 8.0 | 16.0 | 11.0 | 16.0 | 0.0 |
| GAD 7 | 6.0 | 4.0 | 4.5 | -1.5 | 4.5 | 5.0 | 3.0 | -1.5 |
| TSK | 35.5 | 29.0 | 35.5 | 0.0 | 35.5 | 36.5 | 31.0 | -4.5 |
| BPI |  |  |  |  |  |  |  |  |
| Q5 Pain | 3.5 | 5.0 | 4.0 | 0.5 | 4.0 | 4.0 | 4.0 | 0.0 |
| Q6 Pain felt right now | 4.5 | 3.5 | 5.5 | 1.0 | 5.5 | 4.5 | 4.5 | -1.0 |
| Q9 A-G^a^ | 1.8 | 2.1 | 4.4 | 2.6 | 4.4 | 1.8 | 0.9 | -3.4 |
| SF36 |  |  |  |  |  |  |  |  |
| PF | 47.5 | 52.5 | 50.0 | 2.5 | 50.0 | 65.0 | 47.5 | -2.5 |
| RP | 50.0 | 100.0 | 50.0 | 0.0 | 75.0 | 87.5 | 50.0 | -25.0 |
| BP | 57.5 | 40.0 | 57.5 | 0.0 | 62.5 | 57.5 | 57.5 | -5.0 |
| GH | 37.6 | 45.9 | 39.2 | 1.7 | 42.4 | 49.4 | 37.6 | -4.8 |
| VT | 47.5 | 47.5 | 45.0 | -2.5 | 45.0 | 52.5 | 47.5 | 2.5 |
| SF | 75.0 | 75.0 | 81.3 | 6.3 | 75.0 | 81.3 | 75.0 | 0.0 |
| RE | 70.0 | 66.0 | 70.0 | 0.0 | 66.0 | 66.0 | 70.0 | 4.0 |
| MH | 50.0 | 50.0 | 50.0 | 0.0 | 50.0 | 50.0 | 50.0 | 0.0 |
| **Mobility** |  |  |  |  |  |  |  |  |
| 10MWT (sec) | 8.9 | 8.6 | 9.4 | 0.5 | 9.4 | 8.6 | 8.3 | -1.2 |
| 6MWT (m) | 446.0 | 433.0 | 412.0 | -34.0 | 412.0 | 456.0 | 456.0 | 44.0 |
| TUAG (sec) | 8.3 | 8.5 | 7.2 | -1.1 | 7.2 | 7.8 | 8.0 | 0.8 |
| **Muscle strength (lb)** |  |  |  |  |  |  |  |  |
| Right ShoulderAbduction | 34.5 | 34.0 | 33.5 | -1.0 | 33.5 | 33.9 | 34.1 | 0.6 |
| Left Shoulder Abduction | 30.2 | 32.6 | 31.9 | 1.7 | 31.9 | 31.8 | 33.1 | 1.3 |
| Right Hip Flexion^b^ | 18.9 | 21.5 | 24.2 | 5.3 | 24.2 | 27.0 | 22.9 | -1.3 |
| Left Hip Flexion^b^ | 20.4 | 21.5 | 20.8 | 0.5 | 20.8 | 26.1 | 21.9 | 1.1 |
| Right Knee Extension | 44.6 | 43.4 | 44.0 | -0.6 | 44.0 | 52.3 | 51.5 | 7.6 |
| Left Knee Extension | 46.4 | 47.6 | 45.8 | -0.6 | 45.8 | 65.3 | 60.2 | 14.4 |
| Right Hip Abduction | 35.0 | 38.8 | 50.5 | 15.5 | 50.5 | 47.6 | 45.2 | -5.3 |
| Left Hip Abduction | 32.8 | 41.7 | 32.4 | -0.4 | 32.4 | 51.6 | 48.6 | 16.2 |
| Average muscle strength | 32.8 | 35.1 | 35.4 | 2.5 | 35.4 | 41.9 | 39.7 | 4.3 |

^a^ Pain interference measured how much pain has interfered with general activity, mood, walking ability, normal work, relations with other people, sleep and enjoyment of life over the last 24 hours.

*Abbreviations: 6MWT= 6-Minute Walk Test; 10MWT= 10-metre walk test; BPI= Brief Pain Inventory; GAD-7= Generalized Anxiety Disorder 7-item; HRQoL= health-related quality of life; PCS= Pain Catastrophizing Scale; PHQ-9= Participant Health Questionnaire 9-item; S-LANSS= Self-reported- Leeds Assessment of Neuropathic Symptoms and Signs; SF-36= Short Form Health Survey 36-item; TSK-17= Tampa Scale for Kinesiophobia 17-item; TUAG: Timed Up and Go.*

**Supplementary Table 3. Average scores for untreated participants (participants 3 and 4).**

|  | **Hydrotherapy** | | | | **Observation** | | | |
| --- | --- | --- | --- | --- | --- | --- | --- | --- |
|  | **1** | **2** | **3** | **Change during Hydro arm** | **1** | **2** | **3** | **Change during Obs arm** |
| **HRQoL, pain and wellbeing** |  |  |  |  |  |  |  |  |
| PCS | 18.5 | 19.5 | 19.0 | 0.5 | 17.5 | 18.5 | 18.0 | 0.5 |
| PHQ 9 | 7.5 | 7.5 | 8.0 | 0.5 | 9.5 | 8.0 | 7.5 | -2.0 |
| S-LANSS | 5.0 | 1.5 | 0.0 | -5.0 | 0.0 | 2.5 | 0.0 | 0.0 |
| GAD 7 | 10.0 | 5.5 | 3.5 | -6.5 | 6.5 | 3.5 | 5.0 | -1.5 |
| TSK | 34.0 | 41.5 | 36.0 | 2.0 | 37.0 | 36.0 | 35.0 | -2.0 |
| BPI |  |  |  |  |  |  |  |  |
| Q5 Pain | 4.0 | 4.5 | 3.5 | -0.5 | 3.5 | 4.5 | 4.0 | 0.5 |
| Q6 Pain felt right now | 4.0 | 4.0 | 2.5 | 1.5 | 2.0 | 2.5 | 4.0 | 2.0 |
| Q9 A-G^a^ | 18.5 | 20.0 | 15.5 | -3.0 | 19.0 | 12.0 | 21.5 | 2.5 |
| SF36 |  |  |  |  |  |  |  |  |
| PF | 65.0 | 72.5 | 70.0 | 5.0 | 67.5 | 72.5 | 67.5 | 0.0 |
| RP | 50.0 | 100.0 | 50.0 | 0.0 | 75.0 | 87.5 | 50.0 | -25.0 |
| BP | 62.5 | 73.8 | 56.3 | -6.3 | 56.3 | 62.5 | 51.3 | -5.0 |
| GH | 40.1 | 53.4 | 41.7 | 1.7 | 42.4 | 44.4 | 42.6 | 0.2 |
| VT | 27.5 | 45.0 | 37.5 | 10.0 | 32.5 | 42.5 | 40.0 | 7.5 |
| SF | 62.5 | 75.0 | 81.3 | 18.8 | 75.0 | 81.3 | 75.0 | 0.0 |
| RE | 50.0 | 64.0 | 66.0 | 16.0 | 62.0 | 60.0 | 70.0 | 8.0 |
| MH | 50.0 | 50.0 | 50.0 | 0.0 | 50.0 | 50.0 | 50.0 | 0.0 |
| **Mobility** |  |  |  |  |  |  |  |  |
| 10MWT (sec) | 6.5 | 5.9 | 5.7 | -0.8 | 6.2 | 6.1 | 6.2 | -0.1 |
| 6MWT (m) | 496.0 | 562.0 | 558.0 | 62.0 | 553.0 | 542.0 | 539.0 | -14.0 |
| TUAG (sec) | 5.8 | 6.0 | 6.4 | 0.7 | 6.4 | 6.5 | 5.9 | -0.6 |
| **Muscle strength (lb)** |  |  |  |  |  |  |  |  |
| Average muscle strength | 44.1 | 41.7 | 50.4 | 6.4 | 49.6 | 50.8 | 49.0 | -0.6 |

^a^ Pain interference measured how much pain has interfered with general activity, mood, walking ability, normal work, relations with other people, sleep and enjoyment of life over the last 24 hours.

*Abbreviations: 6MWT= 6-Minute Walk Test; 10MWT= 10-metre walk test; BPI= Brief Pain Inventory; GAD-7= Generalized Anxiety Disorder 7-item; HRQoL= health-related quality of life; PCS= Pain Catastrophizing Scale; PHQ-9= Participant Health Questionnaire 9-item; S-LANSS= Self-reported- Leeds Assessment of Neuropathic Symptoms and Signs; SF-36= Short Form Health Survey 36-item; TSK-17= Tampa Scale for Kinesiophobia 17-item; TUAG: Timed Up and Go.*

**Supplementary Table 4. Average scores for participants receiving ERT (participants 1, 2 and 5).**

|  | **Hydrotherapy** | | | | **Observation** | | | |
| --- | --- | --- | --- | --- | --- | --- | --- | --- |
|  | **1** | **2** | **3** | **Change during Hydro arm** | **1** | **2** | **3** | **Change during Obs arm** |
| **HRQoL, pain and wellbeing** |  |  |  |  |  |  |  |  |
| PCS | 9.3 | 7.3 | 1.0 | -8.3 | 1.3 | 3.0 | 2.0 | 0.7 |
| PHQ 9 | 3.0 | 2.0 | 0.0 | -3.0 | 0.0 | 2.0 | 1.3 | 1.3 |
| S-LANSS | 5.0 | 1.5 | 0.0 | -5.0 | 0.0 | 2.5 | 0.0 | 0.0 |
| GAD 7 | 3.3 | 3.7 | 1.0 | -2.3 | 1.0 | 1.0 | 2.7 | 1.7 |
| TSK | 38.3 | 36.0 | 29.7 | -8.7 | 32.0 | 28.7 | 30.7 | -1.3 |
| BPI |  |  |  |  |  |  |  |  |
| Q5 Pain | 3.3 | 4.3 | 3.7 | 0.3 | 3.3 | 7.3 | 5.0 | 1.7 |
| Q6 Pain felt right now | 2.3 | 5.0 | 3.7 | 1.3 | 4.0 | 5.3 | 5.3 | 1.3 |
| Q9 A-G^a^ | 30.7 | 14.7 | 5.7 | -25.0 | 7.3 | 19.3 | 14.0 | 6.7 |
| SF36 |  |  |  |  |  |  |  |  |
| PF | 43.3 | 63.3 | 73.3 | 30.0 | 75.0 | 70.0 | 73.3 | -1.7 |
| RP | 58.3 | 83.3 | 91.7 | 33.3 | 91.7 | 58.3 | 100.0 | 8.3 |
| BP | 52.5 | 60.8 | 75.8 | 23.3 | 79.2 | 60.8 | 75.0 | -4.2 |
| GH | 51.7 | 61.3 | 50.0 | -1.7 | 51.7 | 56.7 | 55.0 | 3.3 |
| VT | 56.7 | 60.0 | 68.3 | 11.7 | 71.7 | 75.0 | 75.0 | 3.3 |
| SF | 62.5 | 83.3 | 87.5 | 25.0 | 87.5 | 70.8 | 79.2 | -8.3 |
| RE | 65.3 | 66.7 | 62.7 | -2.7 | 62.7 | 62.7 | 78.7 | 16.0 |
| MH | 77.8 | 77.8 | 77.8 | 0.0 | 77.8 | 100.0 | 100.0 | 22.2 |
| **Mobility** |  |  |  |  |  |  |  |  |
| 10MWT (sec) | 7.5 | 6.5 | 6.6 | -0.9 | 6.7 | * | 7.1 | 0.4 |
| 6MWT (m) | 451.5 | 509.5 | 518.0 | 66.5 | 513.0 | * | 484.0 | -29.0 |
| TUAG (sec) | 6.4 | 6.3 | 6.4 | 0.0 | 6.6 | * | 6.1 | -0.5 |
| **Muscle strength (lb)** |  |  |  |  |  |  |  |  |
| Average muscle strength | 44.1 | 41.7 | 50.4 | 6.4 | 49.6 | 50.8 | 49.0 | -0.6 |

^a^ Pain interference measured how much pain has interfered with general activity, mood, walking ability, normal work, relations with other people, sleep and enjoyment of life over the last 24 hours.

*Indicates missing data.

*Abbreviations: 6MWT= 6-Minute Walk Test; 10MWT= 10-metre walk test; BPI= Brief Pain Inventory; GAD-7= Generalized Anxiety Disorder 7-item; HRQoL= health-related quality of life; PCS= Pain Catastrophizing Scale; PHQ-9= Participant Health Questionnaire 9-item; S-LANSS= Self-reported- Leeds Assessment of Neuropathic Symptoms and Signs; SF-36= Short Form Health Survey 36-item; TSK-17= Tampa Scale for Kinesiophobia 17-item; TUAG: Timed Up and Go.*
